# Impact of an Interdisciplinary Integrative Group-Based Program for Patients with Cancer: Prospective, Nonrandomized Intervention Study with a Waiting-List Control

**DOI:** 10.3390/curroncol33010044

**Published:** 2026-01-14

**Authors:** Burcu Babadağ Savaş, Bettina Märtens, Yvonne Ziert, Diana Steinmann

**Affiliations:** 1 Department of Radiotherapy, Hannover Medical School, 30625 Hannover, Germany; 2 Klaus–Bahlsen-Center for Integrative Oncology, Comprehensive Cancer Center Lower Saxony, Hannover Medical School, 30625 Hannover, Germany; 3 Institute of Biostatistics, Hannover Medical School, 30625 Hannover, Germany

**Keywords:** integrative oncology, care, quality of life, resilience, oncology, cancer

## Abstract

**Background/Objectives**: Cancer is a disease with a rising global incidence each year, and an interdisciplinary approach for both its treatment and care is needed. This study aimed to evaluate the effects of a 10-week interdisciplinary integrative oncology group-based program for patients with cancer on quality of life, fatigue, resilience, well-being, anxiety and depression. **Methods**: This prospective, nonrandomized intervention, waiting-list control group study evaluated the quality of life, fatigue, resilience, anxiety, depression and well-being of a total of 128 patients with cancer (intervention group: n = 86; waiting-list control group: n = 42) at baseline (week 0) and at the end of the observation period (week 10). **Results**: Compared with patients in the waiting-list group, patients who participated in a 10-week interdisciplinary integrative group program during or after cancer treatment had positive effects on quality of life, social/family well-being, functional well-being, resilience, fatigue, and anxiety. Specifically, significant time × group effects were observed on (FACT-G: *p* = 0.002, *η*^2^ = 0.73; FACIT-Fatigue: *p* = 0.014, *η*^2^ = 0.47; FACIT-F: *p* = 0.002, *η*^2^ = 0.74), social/family well-being (*p* = 0.015, *η*^2^ = 0.46), functional well-being (*p* < 0.001, *η*^2^ = 0.102), with a large effect size and resilience mean scores (*p* = 0.003, *η*^2^ = 0.069), and anxiety mean scores (*p* = 0.005, *η*^2^ = 0.060), with a medium effect size. **Conclusions**: This study revealed that compared with nonparticipants, participants in the 10-week interdisciplinary program benefited more from the program.

## Simple Summary

Patients with cancer struggle with many symptoms during or after cancer treatment, and their quality of life is negatively affected. This study evaluates the effects of an integrative group-based program (exercise, nutrition, meditation, relaxation, aromatherapy, etc.) provided by a 10-week interdisciplinary team. A total of 128 patients participated in either the intervention group or the waiting list-control group. The results of the study revealed improvements in symptoms such as quality of life, resilience, fatigue, and anxiety, particularly among patients who participated in the group program. This study suggests that integrative programs are beneficial for patients and recommends planning randomized controlled trials in the future.

## 1. Introduction

The global incidence of cancer is increasing each year and patients with cancer, particularly during the diagnosis, treatment, and posttreatment phases, face a wide range of challenges that negatively impact their quality of life—including physical and emotional difficulties [1,2]. Additionally, they are trying to cope with various symptoms, such as fatigue, anxiety, and depression [3]. In coping with these situations, individuals’ resilience is also important [4].

Especially in coping with all these symptoms, increasing resilience, and improving quality of life, various complementary and alternative methods may also be needed [5,6]. Many patients with cancer, especially in Germany, use complementary alternative medicine (CAM) and integrative approaches to cope with all these symptoms and increase their resilience [5,6]. Various guidelines also suggest that the use of integrative practices during or after cancer treatment is beneficial, particularly for improving quality of life and functional status and symptom management [7,8,9].

In addition, an interdisciplinary approach is needed for both its treatment and care in order to cope with these symptoms and improve quality of life [1]. Research has indicated that integrative interventions are especially effective when they are delivered by interdisciplinary teams specializing in this field. In Germany, for example, hospitals in cities such as Essen [10] and Berlin [11] have established routine clinical practices that incorporate integrative oncology approaches for cancer patients. Similarly, since 2018, our hospital has implemented a structured “10-week interdisciplinary integrative group program” as part of its daily oncology care [12].

The outcomes of this group program [13], as well as studies examining its long-term effects, have previously been published [6]. However, one major limitation is the absence of a comparison with a control group [13]. Furthermore, the literature includes only a limited number of studies evaluating such integrative interventions in a controlled design [14,15,16].

Therefore, the present study aims to assess the effects of an interdisciplinary integrative, group-based program by comparing it with a waiting-list control group—specifically in terms of its impact on quality of life, fatigue, resilience, anxiety, depression and well-being in patients with cancer.

## 2. Materials and Methods

### 2.1. Study Design

This was a prospective, nonrandomized intervention study with a waiting-list control group. This study evaluated the effects of a 10-week interdisciplinary integrative group-based program on quality of life, fatigue, well-being, anxiety, and depression among patients with cancer. The outcomes were compared with those of a control group on the waiting list. The study procedure and interdisciplinary group-based program were conducted at the Department of Radiotherapy at Hannover Medical School (MHH), within the framework of the Klaus–Bahlsen Center for Integrative Oncology, Comprehensive Cancer Center Hannover (CCC-H).

### 2.2. Participants

This study was conducted between May 2021 and March 2025. Participants who met the inclusion criteria were nonrandomly assigned to either the group program or the waiting list, on the basis primarily of the order of application and individual considerations such as personal needs, appointments, and holidays. Eligible participants were *(1) adults aged 18 or older, (2) those who were currently undergoing or having completed cancer treatment, (3) those who were diagnosed with any type of cancer, (4) those who provided consent to participate in the study, (5) those who had adequate mental and physical capacity, and (6) those who had sufficient proficiency in the German language to participate in the group sessions*. Individuals with severe mental or neurological impairments or those who declined to participate in the survey were excluded from the study.

The sample size of the study was determined based on previously published studies using similar methodologies [13,15]. No formal pre-power analysis was conducted. However, effect sizes were specifically included in all preliminary analyses and were used in interpreting the findings.

### 2.3. Intervention

#### 2.3.1. Interdisciplinary Integrative Oncology Group Program

The group program is conducted by an interdisciplinary team once a week for five hours and consists of a total of 10 sessions, totaling 50 h [6,13,17]. This program was developed on the basis of the mind–body medicine “Essen Model”, which was specifically developed by Dobos and colleagues and whose results have been published [18]. The group program begins in the mornings with small physical exercises performed together and includes a wide range of integrative practices, such as mind–body medicine techniques, nutrition, self-care, aromatherapy, stress management, and social interaction before lunch. In the afternoons, it also covers topics such as creative activities, yoga, dance therapy, klang meditation and Nordic walking. These sessions are led by professionals specializing in their respective fields, including nurses, physicians, mind–body-medicine therapists, nutritionists, psychologists, and various other therapists. Specifically, the 10- week interdisciplinary integrative group program is coordinated by a core team consisting of three physicians, two nurses, one nutritionist, and one program coordinator responsible for scheduling and organizational tasks. Additional creative sessions are conducted by specialized therapists such as yoga, dance therapy or klang mediation. All team members maintain their regular clinical responsibilities alongside program duties, and the interdisciplinary team the program is funded by the Rut und Klaus Bahlsen Foundation.

On average, 12 participants join each cycle of the group program. Initially established in 2018 at the Department of Radiotherapy at MHH, the program was provided since 2022 by the Klaus Bahlsen Center for Integrative Oncology, CCC-H. The program is free of charge and open to all patients with cancer. It is currently ongoing [17], and initial outcomes have already been published [6,13]. The Rut und Klaus Bahlsen Stiftung provides funding; no health insurance coverage is available for the program.

Program information and announcements are shared through the official website [17], brochures, journals, and posters and via the complementary medicine consultation hours provided by physicians and nurses at the center. Patients who are interested in participating contact the center and are first offered consultation with a physician from the interdisciplinary team. Patients were evaluated for eligibility by the interdisciplinary team, and those considered suitable and willing to participate were enrolled in the group program through a nonrandomized allocation process.

#### 2.3.2. Intervention Group

Patients who participated in both the group program and the study formed the intervention group. These patients were assessed using relevant questionnaires at baseline (week 0) and at the end of the program (week 10). Priority for program admission was given on the basis of the order of application and personal circumstances (e.g., important appointments, holidays).

#### 2.3.3. Waiting-List Control Group

Patients who expressed interest in the group program but had a waiting period of at least 10 weeks were placed on a waiting list. Those who agreed to participate in the study were assessed using the same questionnaires at week 0 and week 10. After completing the assessment period, they were then enrolled in the group program sequentially.

### 2.4. Data Collection and Measurements

All assessments were conducted using the questionnaires detailed below, either via the online platform SoSci Survey or in paper format.

#### 2.4.1. The Functional Assessment of Cancer Therapy–General (FACT-G)

The Functional Assessment of Cancer Therapy-General (FACT-G) instrument was developed specifically to measure health-related quality of life (HRQOL) in patients with cancer. The German version was used in this study. This instrument consists of a total of 27 items and is a 5-point Likert scale (0–4). It consists of 4 subscales: these are physical well-being (PWB), social/family well-being (SWB), emotional well-being (EWB), and functional well-being (FWB). The sum of all subscales constitutes the FACT-G score. Higher scores for the scale and subscales indicate a better quality of life [15,19].

#### 2.4.2. The Functional Assessment of Chronic Illness Therapy–Fatigue Scale (FACIT-Fatigue)

In addition to the FACT-G, we used the FACIT-Fatigue to specifically assess cancer-related fatigue and its impact on daily life and functioning. This subscale includes 13 items, which are also rated on a 5-point Likert scale (0–4), and it complements the FACT-G by focusing on fatigue-related aspects of well-being [20].

#### 2.4.3. Resilience Scale (RS-13)

The resilience scale consists of a total of 13 questions (ranging from 1 to 7), and the German version was used in this study. Higher scores indicate better resilience. Scores between 13 and 66 indicate low resilience, scores between 67 and 72 indicate medium resilience, and scores between 73 and 91 indicate high resilience [21].

#### 2.4.4. Hospital Anxiety and Depression Scale (HADS-D)

The HADS-D consists of a total of 14 items (ranging from 0 to 3) used to measure anxiety and depression in patients with physical illnesses. The German version was used in this study. There are two subscales: anxiety and depression. Higher scores indicate more severe distress—patients can be classified according to their individual sum scores: non-case (0–7), borderline case (8–10) and definite case (11 and above) [22,23].

#### 2.4.5. The World Health Organization-Five Well-Being Index (WHO-5)

The German version of the WHO-5 was used to measure mental well-being. It consists of 5 items rated on a 6-point Likert scale (0–6). Higher scores indicate better mental well-being [24].

### 2.5. Statistical Analysis

Statistical analyses were performed using IBM SPSS Statistics (Statistical Package for Social Sciences Version 29.0.1.1). The normal distribution of the metric data was evaluated using the Kolmogorov–Smirnov test and a histogram. Descriptive analyses (means, standard deviations, absolute and relative frequencies) were used to describe the data. Pearson’s chi-square test, Student’s *t* test and Fisher’s test were employed to compare patient characteristics between the two groups. Repeated measures of quality of life, fatigue, resilience, anxiety, depression and well-being scores, assessed using the FACT-G, FACIT-Fatigue, RS-13, HADS-D, and WHO-5 Index, were analyzed and compared between groups using one-way repeated-measures ANOVA. Eta-square (*η*^2^) effect sizes were calculated to express the mean score differences in groups across the measurement times. Effect sizes were defined as follows: *η*^2^ ≥ 0.01, small effect; *η*^2^ ≥ 0.06, medium effect; and *η*^2^ ≥ 0.14, large effect [25].

The number of patients varies in the statistical analysis because SPSS drops cases with missing values by default. Due to the non-confirmative character of the study design, *p*-values are only interpreted in a descriptive way and were not adjusted for multiplicity. However, *p* values smaller than 0.05 were considered as indicating statistical significance.

### 2.6. Ethical Consideration

This study was approved by the ethics committee of the university (Approval number: 8204_BO_S_2018; Approval date: 7 May 2021). Informed consent was obtained from all participants, either through an online form or via a paper-and-pencil format, depending on individual preference and accessibility. This study was reported in accordance with the STROBE guidelines [26].

## 3. Results

**Figure 1:**
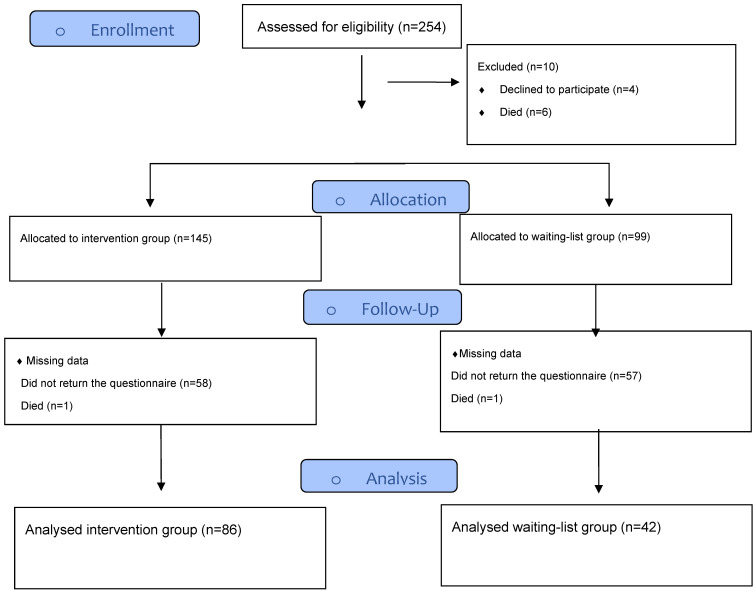
Patient flow diagram.

A total of 145 patients were allocated in the group program, and 99 patients were placed on the waiting list. Among these, 128 patients who met the eligibility criteria, provided informed consent, and completed the questionnaires at baseline (week 0) and at the end of the observation period (week 10) were included in the final per-protocol analysis (Figure 1). Of these patients, 86 were assigned to the intervention group, while 42 were nonrandomized and allocated to the waiting-list control group.

### 3.1. Patient Characteristics (Sociodemographic and Medical)

The mean ages of the patients were 56.74 ± 11.18 years in the intervention group and 57.45 ± 9.78 years in the waiting-list group. In both groups, the majority of patients were female, had an intermediate level of education, and were employed. In the intervention and control groups, most patients had been diagnosed with breast cancer (77.4% vs. 73.8%), and the majority were at a primary stage of diagnosis (80.2% vs. 85.7%), with more than half having completed cancer treatment (53.5% vs. 59.5%). Compared with the waiting-list group (45.2%), a significantly larger proportion of the intervention group (68.6%) had completed cancer treatment before the group program (*p* = 0.013). In the intervention group, 54.7% of patients received cancer treatment (hormone therapy (25.6%), other therapies (18.6%), radiotherapy (10.5%) and/or chemotherapy (10.5%) during the group program. In the waiting-list group, 78.6% of patients received cancer treatment (radiotherapy (38.1%), chemotherapy (33.3%), hormone therapy (28.6%), or other therapies (19.0%)) during the waiting period of 10 weeks (*p* = 0.011). Most patients indicated a strong interest in topics such as awareness of feelings (52.3%), nutrition (50.3%) and voices within us (the inner child) (46.9%). Both groups were similar in terms of patient characteristics, such as age, sex, education level, employment status, and cancer diagnosis (*p* > 0.05), except for having undergone cancer treatment before or during the group program or while on the waiting list (*p* < 0.05) (Table 1).

### 3.2. Comparison of FACT-G and FACIT-Fatigue Scores at Weeks 0 and 10

**Figure 2:**
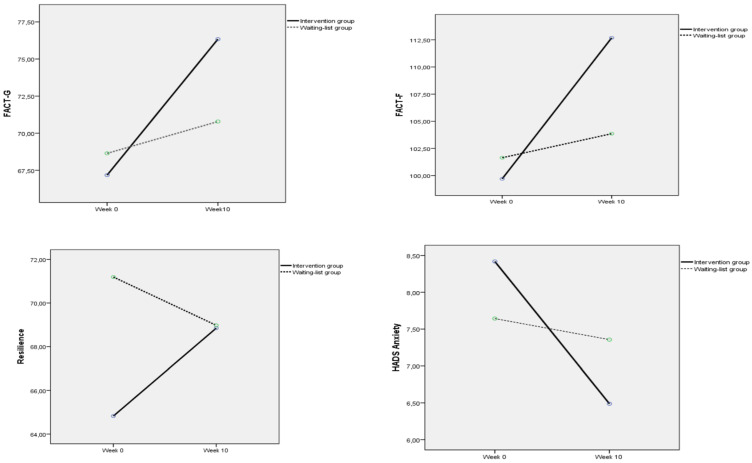
Changes for FACT-G, FACIT-F, Resilience and HADS Anxiety Mean Scores at Week 0 and Week 10. Abbreviations: FACT-G = Functional Assessment of Cancer Therapy—General, HADS = Hospital Anxiety and Depression Scale.

From week 0 to week 10, the intervention group showed significant improvements across all three scales—FACT-G (from 67.17 ± 13.55 to 76.34 ± 17.25), FACIT-Fatigue (from 32.52 ± 11.95 to 36.35 ± 11.60), and FACIT-F (from 99.70 ± 24.03 to 112.69 ± 27.96)—while the waiting-list group exhibited only minor changes [FACT-G (from 68.64 ± 13.46 to 70.79 ± 17.88), FACIT-Fatigue (from 33.00 ± 13.11 to 33.07 ± 13.17), and FACIT-F (from 101.64 ± 25.38 to 103.86 ± 30.35)]. These improvements were supported by significant time × group interactions with large effect sizes (FACT-G: *p* = 0.002, *η^2^* = 0.73; FACIT-Fatigue: *p* = 0.014, *η^2^* = 0.47; FACIT-F: *p* = 0.002, *η^2^* = 0.74), indicating that the intervention group improved substantially more than the waiting-list group did over the 10 weeks (Table 2, Figure 2).

When week 0 and week 10 were compared, the mean scores for *social/family well-being* and *functional well-being* improved in the intervention group but decreased in the waiting-list group. Notably, a significant and large time × group interaction effect with a large effect size was observed (SWB: *p* = 0.015, *η^2^* = 0.46; FWB: *p* < 0.001, *η^2^* = 0.102). In contrast, *physical well-being* and *emotional well-being* in the intervention group increased only slightly compared with those in the waiting-list group at week 10. Although a significant main effect of time was detected with a large effect size (both *p* < 0.001, *η^2^* > 0.14), time × group interaction effects were observed (both *p* > 0.05, *η^2^* > 0.01) (Table 2).

### 3.3. Comparisons of the Resilience, HADS, and Well-Being Scores at Weeks 0 and 10

The mean resilience scores of patients in the intervention group increased from 64.82 ± 15.72 at week 0 to 68.85 ± 13.85 at week 10, moving from the low-resilience category to the medium-resilience category. In contrast, patients in the waiting-list control group demonstrated a decrease in their mean resilience score, from 71.19 ± 14.54 (medium-resilience) at week 0 to 68.98 ± 16.24 (medium-resilience) at week 10. Statistical analysis revealed that the time × group interaction was significant (*p* = 0.003, *η^2^* = 0.069), indicating a medium effect size. These findings indicate that patients in the intervention group experienced an improvement in resilience through participation in the integrative group program (Table 3, Figure 2).

The mean total HADS score at week 0 and week 10 was greater in the intervention group (from 15.26 ± 8.52 to 11.52 ± 7.82) than in the waiting-list group (from 14.17 ± 8.19 to 13.12 ± 7.44). Statistical analysis revealed a significant main effect of time with a large effect size (*p* < 0.001, *η^2^* = 0.141), indicating an overall improvement in HADS scores. The between-group effect was also significant, although with a small effect size (*p* = 0.012, *η^2^* = 0.049). However, the time × group interaction was not significant (*p* = 0.859, *η^2^* = 0.000), suggesting that improvements in HADS scores were observed in both groups, without differential effects between time and group (Table 3).

In terms of mean *anxiety* scores, a more pronounced improvement was observed in the intervention group than in the waiting-list group at the end of week 10. Patients in the intervention group moved from the borderline category (mean 8.41 ± 4.40 at week 0) to the non-case category (mean 6.49 ± 4.22 at week 10). In contrast, the waiting-list group showed only a slight change, from 7.64 ± 4.40 at week 0 to 7.35 ± 3.81 at week 10. Statistical analysis indicated that the time × group interaction was significant, with a medium effect size (*p* = 0.005, *η^2^* = 0.060), suggesting that the observed improvement over time was strongly associated with participation in the intervention program (Table 3, Figure 2).

Although improvement was observed in the mean *depression* scores in both groups at the end of week 10 (intervention group: from 6.83 ± 4.94 to 5.03 ± 4.36; waiting-list group: from 6.52 ± 4.35 to 5.76 ± 4.20), the mean time effect was statistically significant and had a large effect size (*p* < 0.001, *η^2^* = 0.128). However, the interaction between group (*p* = 0.797, *η^2^* = 0.001) and time × group is not significant (*p* = 0.083, *η^2^* = 0.024) (Table 3).

With respect to the mean WHO-5 score, greater improvement in mental well-being was observed in the intervention group (from 11.85 ± 5.80 at week 0 to 14.56 ± 5.82 at week 10) than in the waiting-list group (from 12.40 ± 6.44 to 12.57 ± 6.15). Statistical analysis revealed a significant main effect of time with a medium effect size (*p* < 0.001, *η^2^* = 0.097) and a significant between-group effect (*p* = 0.001, *η^2^* = 0.077). However, the time × group interaction was not significant (*p* = 0.499, *η^2^* = 0.004), indicating that while overall mental well-being improved, the group program did not produce a differential effect on WHO-5 scores over time between groups (Table 3).

### 3.4. Safety and Adherence

No serious adverse events were reported by any of the patients. Approximately 10% of the participants reported feeling overwhelmed at certain points during the course. Reported challenges included listening to other patients’ illness histories, experiencing emotional difficulties when addressing inner-child work, and difficulties related to specific activities such as yoga and qigong, including problems with maintaining balance or physical activity during classes. Overall, 85% of the patients (n = 73) participated in the full-day group program, and among these, 88% participated for five or more days.

## 4. Discussion

This study investigated changes in quality of life, fatigue, resilience, anxiety, depression, and well-being among patients with cancer during or after treatment and before and after their participation in a 10-week integrative group-based program and compared the results with those of a waiting-list control group. In our study, some improvements were observed in the intervention group compared with the waiting-list group in terms of overall quality of life and fatigue, as measured on the FACT-G, FACIT-F, and FACIT-Fatigue scales between week 0 and week 10, with a time × group interaction and a large effect size. Furthermore, while some improvements were found in social/family well-being and functional well-being in the intervention group, the waiting-list group showed decreases. In the literature, mind–body-medicine interventions during or after cancer treatment positively affect patients with cancer. In the study by Jeiter et al., a 12-week evaluation of a mind–body medicine-based day care clinic revealed a significant change in quality of life compared with the waiting-list group [15], similar to our study. Furthermore, in our previously published study [13], which evaluated the effectiveness of the interdisciplinary integrative group-based program without a control group, increases in the quality of life and resilience scores of patients with cancer were observed after the group program.

Patients who participated in the group program showed improvement in resilience, moving from the low-resilience category to the medium-resilience category after the group program, whereas the waiting-list group showed almost no change. The group program in particular, improved patients’ resilience. Similar to the results of our study, in a previous study examining the long-term effects of the interdisciplinary group-based program, resilience increased significantly ≥12 months after participation in the 10-week group program, with a medium effect size. Additionally, patients continued to use complementary and alternative medicine practices, especially from mind–body medicine, even 1 to 4.5 years after completing the program [6]. In Li Oei et al.’s study, patients with breast cancer were followed up for up to 12 months after their initial diagnosis at a breast cancer center, where integrative practices (nursing compresses, music therapy, rhythmic massage, etc.) were integrated alongside standard oncological treatment. After 12 months, an increase in patients’ inner resilience and coherence was observed. The integration of interdisciplinary multimodal programs into routine clinical practice in cancer patient care may positively impact patient care [27]. In addition, in an Asher et al. randomized, waiting list-controlled study evaluating the effect of a 6-week strengthening program on resilience in women with metastatic cancer, statistically significant improvements in psychological well-being and overall quality of life (mean scores of FACT-G) were observed compared with those in the waiting-list control group. Furthermore, improvements in depression, loneliness, and anxiety were observed in the follow-up of participants who participated in the program. As a result, studies in the literature comparing the control group or only measuring the effectiveness of the group program revealed significant changes in variables such as general well-being, quality of life, resilience, loneliness, and anxiety [16]. These findings suggest that supporting patients with cancer via integrative multidisciplinary holistic programs during and after treatment, ensuring that they are not alone, will lead to significant improvements in their fight against the disease and in patient outcomes.

Additionally, in our study, while greater improvement was observed in HADS scores, particularly in the intervention group, a significant effect at a medium effect level was observed, especially in the anxiety subscale, compared with the waiting-list group. In addition, no significant changes in depression or well-being mean scores were observed over time in either group. The American Society of Clinical Oncology (ASCO) and the Integrative Oncology Society updated guidelines on managing anxiety, depression, and fatigue and the application of integrative approaches in patients with cancer. Strong recommendations are made for mindfulness-based stress reduction, mindfulness-based cognitive therapy, and Tai chi or qigong for managing cancer fatigue during treatment and for mindfulness-based programs for managing cancer fatigue after cancer treatment. Methods recommended for managing anxiety include mindfulness-based interventions such as yoga, relaxation therapies, music therapy, or lavender essential oils [8]. Chang et al. investigated the effectiveness of an integrated 8-week mindfulness-based fitness training program compared with a waiting-list control group in breast cancer patients and reported that the program had a positive effect, particularly on improving patients’ well-being and reducing their fear of recurrence [14]. Additionally, in another study by Mao et al., a randomized controlled trial investigating the effectiveness of a 12-week digital integrative medicine program in patients undergoing treatment for solid tumors revealed that those using the program experienced a reduction in fatigue, anxiety, depression and distress compared to those receiving usual care [28]. Therefore, mind–body medicine-based programs are crucial for managing symptoms related to disease and treatment in cancer patients, both during and after treatment.

Furthermore, the majority of the study population consisted of female patients. As observed in our previous studies, female patients showed greater interest in participating in the group program [6,13,15]. This predominance may be explained by the more widespread use of CAM and integrative approaches among women, their more frequent participation in health-related programs, a higher motivation to seek psychosocial support, and gender-related differences in coping behaviors [6,29].

In particular, patients who participated in the group program showed improvements in quality of life, fatigue, anxiety, and resilience. Although the basic sociodemographic data were statistically similar in both groups, compared with patients on the waiting list, patients in the intervention group completed their cancer treatment before the group program, and there was a difference between the two groups. This situation is thought to be because patients in the waiting-list group either wanted to complete their treatment before they participated in the group program or were obliged to do so. Nevertheless, despite the intervention group containing fewer patients undergoing active treatment, these observed changes are still significant, and the group program may have contributed to this.

### Limitations

Our study has several limitations. Although our study included a waiting-list control group, the study design was nonrandomized. In addition, some differences in patient characteristics, such as receiving treatment during the group program or waiting period, were observed between the two groups. Furthermore, the statistical analysis is based on the per-protocol population (patients who participated in data capture at both timepoints and who participated in the entire intervention), which may be the most adherent patients. This situation may affect the study outcomes in a positive way. This study did not assess the long-term effects of the group program on other daily activities outside of the group program.

## 5. Conclusions

Patients with cancer cope with multiple symptoms during and after treatment, and integrative approaches provided by an interdisciplinary team may play an important role in improving quality of life and resilience and in managing symptoms. In this study, compared with patients in the waiting-list group, patients who participated in a 10-week interdisciplinary integrative group program during or after cancer treatment experienced positive effects on quality of life, social/family well-being, functional well-being, resilience, fatigue, and anxiety. These findings suggest that such programs delivered by interdisciplinary teams may represent a valuable supportive care option for patients during and after cancer treatment. It is recommended that future studies be conducted in randomized controlled groups and in combined studies that also examine the effects in later periods to further evaluate the effectiveness and sustainability of these interventions.

## Figures and Tables

**Table 1:** Patient Characteristics of participants (N = 128).

Characteristic	Intervention Group		Waiting-List Group		Statistical Analysis
	n	Mean ± SD	n	Mean ± SD	*p*
**Age (yr)**	86	56.74 ± 11.18	42	57.45 ± 9.78	0.727 ^a^
	**n**	**%**	**n**	**%**	***p***
**Gender**					
Female	80	93.0	40	95.2	0.1000 ^b^
Male	6	7.0	2	4.8	
**Education level**					0.384 ^c^
Lower secondary school	2	2.3	0	0.0	
Advanced technical certificate	17	19.8	13	31.0	
Intermediate school	57	66.3	26	61.9	
High school	10	11.6	3	7.1	
**Employment status**					0.620 ^c^
Employed	33	38.4	20	47.6	
Retired	26	30.2	14	33.3	
Housemaker/Unemployed	5	5.8	1	2.4	
Others	22	25.6	7	16.7	
**Cancer diagnosis**					0.774 ^c^
Breast cancer	64	77.4	31	73.8	
Colorectal cancer	3	3.5	3	7.1	
Gynecologic cancer	3	3.5	2	4.8	
Prostate cancer	2	2.3	0	0.0	
Others *	14	16.3		14.3	
**Cancer diagnosis stage**					0.672 ^c^
Primary	69	80.2	36	85.7	
Residual	9	10.5	4	9.5	
Metastatic	8	9.3	2	4.8	
Cancer treatment received before group program ***					
No	27	31.4	23	54.8	**0.013 ^c^**
Yes	59	68.6	19	45.2	
Radiotherapy	40	46.5	15	35.7	0.113 **^c^**
Chemotherapy	33	38.4	6	14.3	**0.016 ^c^**
Surgery	50	58.1	16	38.1	0.088 **^c^**
Hormone therapy	10	11.6	2	4.8	0.093 **^c^**
Others **	6	7.0	1	2.4	0.120 **^b^**
Cancer treatment during group program or waiting ***					
No	39	45.3	9	21.4	**0.011 ^c^**
Yes	47	54.7	33	78.6	
Radiotherapy	9	10.5	16	38.1	**<0.001 ^c^**
Chemotherapy	9	10.5	14	33.3	**0.002 ^c^**
Surgery	2	2.3	6	14.3	**0.004 ^b^**
Hormone therapy	22	25.6	12	28.6	**0.020 ^c^**
Others **	16	18.6	8	19.0	**0.020 ^c^**

^a^ Student’s *t*-test, ^b^ Fischer Test, ^c^ Pearson Chi-Square Test. * Others (esophageal and gastric cancer, kidney cancer, bladder cancer, neuroendocrine tumor, skin cancer, etc.). ** Others (immunotherapy, antihormone therapy etc.). *** More than 1 option could be marked by the patient in the questionnaire.

**Table 2:** Comparison of FACT-G and FACIT-Fatigue Mean Scores at weeks 0 and 10.

Scores	Group	Week 0	Week 10	Time		Between Group		Time × Group	
		Mean ± SD n = 86	Mean ± SD n = 42	*p*-Value *	*η* ^2^	*p*-Value *	*η* ^2^	*p*-Value *	*η* ^2^
**FACT-G**	Intervention Group	67.17 ± 13.55	76.34 ± 17.25	**<0.001**	**0.17**	0.454	0.004	**0.002**	**0.73**
	Waiting-list Group	68.64 ± 13.46	70.79 ± 17.88						
***Physical Well-Being (PWG)***	Intervention Group	19.20 ± 5.72	21.49 ± 5.26	**<0.001**	**0.96**	0.187	0.14	0.083	0.024
	Waiting-list Group	18.64 ± 5.85	19.45 ± 6.18						
***Social/Family Well-Being (SWB)***	Intervention Group	19.28 ± 5.63	21.49 ± 5.26	0.265	0.10	0.533	0.003	**0.015**	**0.46**
	Waiting-list Group	20.28 ± 4.64	19.45 ± 6.18						
***Emotional Well-Being (EWB)***	Intervention Group	13.40 ± 2.87	17.53 ± 4.48	**<0.001**	**0.253**	0.525	0.003	0.459	0.004
	Waiting-list Group	13.55 ± 3.49	16.83 ± 4.28						
***Functional Well-Being (FWB)***	Intervention Group	15.27 ± 5.60	17.53 ± 5.70	0.092	0.22	0.509	0.003	**<0.001**	**0.102**
	Waiting-list Group	16.17 ± 6.01	15.30 ± 6.16						
***FACIT-Fatigue***	Intervention Group	32.52 ± 11.95	36.35 ± 11.60	**0.011**	**0.50**	0.521	0.003	**0.014**	**0.47**
	Waiting-list Group	33.00 ± 13.11	33.07 ± 13.17						
**FACIT-F**	Intervention Group	99.70 ± 24.03	112.69 ±27.96	**<0.001**	**0.138**	0.468	0.004	**0.002**	**0.74**
	Waiting-list Group	101.64 ± 25.38	103.86 ± 30.35						

Abbreviations: FACT-G = Functional Assessment of Cancer Therapy—General, FACIT-Fatigue = Functional Assessment of Chronic Illness Therapy—Fatigue, n = numbers of patients, SD = Standard deviation. * Repeated-Measures ANOVA; bold *p*-values indicate significant differences between time points (*p* < 0.05).

**Table 3:** Comparison of Resilience, HADS and WHO-5 Mean Scores at week 0 and 10.

Scores	Group	Week 0	Week 10	Time		Between Group		Time × Group	
		Mean ± SD n = 86	Mean ± SD n = 42	*p*-Value *	*η* ^2^	*p*-Value *	*η* ^2^	*p*-Value *	*η* ^2^
**Resilience**	Intervention Group	64.82 ± 15.72	68.85 ± 13.85	0.375	0.006	0.221	0.12	**0.003**	**0.069**
	Waiting-list Group	71.19 ± 14.54	68.98 ± 16.24						
**HADS**	Intervention Group	15.26 ± 8.52	11.52 ± 7.82	**<0.001**	**0.141**	**0.012**	**0.049**	0.859	0.000
	Waiting-list Group	14.17 ± 8.19	13.12 ± 7.44						
***Anxiety***	Intervention Group	8.41 ± 4.40	6.49 ± 4.22	**<0.001**	**0.104**	0.950	0.000	**0.005**	**0.060**
	Waiting-list Group	7.64 ± 4.40	7.35 ± 3.81						
***Depression***	Intervention Group	6.83 ± 4.94	5.03 ± 4.36	**<0.001**	**0.128**	0.797	0.001	0.083	0.024
	Waiting-list Group	6.52 ± 4.35	5.76 ± 4.20						
**WHO-5**	Intervention Group	11.85 ± 5.80	14.56 ± 5.82	**<0.001**	**0.097**	**0.001**	**0.077**	0.499	0.004
	Waiting-list Group	12.40 ± 6.44	12.57 ± 6.15						

Abbreviations: HADS = Hospital Anxiety and Depression Scale, WHO-5 = The World Health Organization-Five Well-Being Index, n = numbers of patients, SD = Standard deviation, * Repeated-Measures ANOVA; bold *p*-values indicate significant differences between time points (*p* < 0.05).

## Data Availability

The datasets used and/or analyzed during the current study are available from the corresponding authors upon reasonable request.
